# Health professionals’ experiences and perspectives on food insecurity and long‐term conditions: A qualitative investigation

**DOI:** 10.1111/hsc.12872

**Published:** 2019-10-08

**Authors:** Flora Douglas, Kathryn Machray, Vikki Entwistle

**Affiliations:** ^1^ School of Nursing and Midwifery Robert Gordon University Aberdeen UK; ^2^ MRC/CSO Social and Public Health Sciences Unit University of Glasgow Glasgow UK; ^3^ Centre for Biomedical Ethics National University of Singapore Singapore Singapore

**Keywords:** clinical management, food insecurity, long‐term health conditions, self‐management support

## Abstract

Estimates suggest that over 10% of the UK population are affected by food insecurity. International evidence indicates that food insecurity is a risk factor for many long‐term health conditions, and can adversely affect people's ability to manage existing conditions. Food insecurity is thus not only a serious social concern but also a healthcare issue requiring the attention of UK health professionals. An exploratory qualitative study was undertaken to investigate the experiences and views of health professionals in north east Scotland, with a particular focus on support for people with long‐term conditions whom they believed were affected by food insecurity. Two focus groups and nine semi‐structured interviews were undertaken with a total of 20 health professionals between March and July 2016. Thematic analysis generated three main themes. The health professionals had (a) *diverse levels of understanding and experience of food insecurity,* but between them identified a range of (b) *negative impacts of food insecurity on condition‐management,* especially for diet dependent conditions or medication regimes, and for mental health. Even for those health professionals more familiar with food insecurity, there were various (c) *practical and ethical uncertainties about identifying and working with food insecure patients* (it could be difficult to judge, for example, whether and how to raise the issue with people, to tailor dietary advice to reflect food insecurity, and to engage with other agencies working to address food insecurity). This study indicates that health professionals working with food insecure patients have learning and support needs that warrant further investigation. Debates about health professionals’ responsibilities, and interventions to guide and support health professionals, including tools that might be used to screen for food insecurity, must also reflect the diverse lived needs and values of people who experience food insecurity.

**What is known about this topic**

- Household food insecurity is a risk factor for various chronic health conditions and can compromise self‐management of existing conditions.
- Little is known about how health professionals in the UK experience work with food insecure patients.

**What this study adds**

- The extent to which health professionals are aware of food insecurity, either in general terms or in particular patients, varies greatly.
- Health professionals can experience practical and ethical uncertainty about how to identify and respond to food insecurity among their patients.

## 1 INTRODUCTION

Food insecurity is an indication of economic struggle and a serious healthcare issue (Chilton, Knowles, & Bloom, 2017; Gundersen & Ziliak, 2018; Patil, Craven, & Kolasa, 2017; Scottish Government, 2015). Food insecurity arises when a person or household has inadequate or insecure access to food due to financial or other constraints (Seligman, Davis, Schillinger, & Wolf, 2010). It is increasingly recognised as a problem in low‐income households in high‐income countries (Lambie‐Mumford, 2019; Loopstra, Reeves, & Tarasuk, 2019; Reeves, Loopstra, & Stuckler, 2017). Food insecurity exists on a continuum from ‘milder’ manifestations such as uncertainty and anxiety about food access, through insufficient access to appropriate types and amounts of food, to more severe experiences involving hunger (Kendall, Olson, & Frongillo, 1995). Being food insecure may be exacerbated by, and also increase the risk of social isolation or exclusion as it constrains participation in social and cultural activities associated with food (Healy, 2019; Meijs, Handy, Simons, & Roza, 2019).

Food insecurity is associated with increased risk of serious non‐communicable health conditions including cancer, diabetes, cardiovascular disease (Gundersen & Ziliak, 2015). It also compromises health condition‐management, leading to sub‐optimal health outcomes (Gucciardi, Vahabi, Norris, Del Monte, & Farnum, 2014; Laraia, 2013; Seligman et al., 2010; Vozoris & Tarasuk, 2003). In North America it has also been independently associated with increased healthcare use and costs (Berkowitz, Basu, Meigs, & Seligman, 2018; Tarasuk et al., 2015).

Just over 10% of the UK population live in food insecure households and projections point towards a worsening picture (Josepeh Rowntree Foundation, 2018). The probability of low‐income UK households being food insecure rose from 28% in 2004 to 46% in 2016, with disabled people living in such circumstances shown to be particularly badly affected (Loopstra et al., 2019). Over 42% of patients registered with a GP in Scotland live with one or more long‐term condition (Barnett et al., 2012). Long‐term conditions are estimated to account for 70% of health and social care spending in England, and 80% of GP consultations in Scotland (Department of Health, 2010; Iacobucci, 2017; Scottish Government, 2015). Moreover, people affected by health conditions are amongst the highest users of food banks in the UK (Garthwaite, Collins, & Bambra, 2015; Loopstra & Lalor, 2017), and the intersection of low‐income and debt with ill‐health or disability increases both the challenges of access to food and the risk of destitution (Fitzpatrick, Bramley, Sosenko, & Blenkinsopp, 2018). Health professionals have become informal referral agents to food banks (Douglas et al., 2018), but food banks cannot provide either the quantity or quality of food suitable for good health condition‐management (Loopstra et al., 2019; Thompson, Smith, & Cummins, 2018). Seligman and Berkowitz (2018) suggest that not only does constrained food access impact people's ability to cope with their condition, but the associated uncertainty and anxiety leads to (mal)adaptive coping strategies and sub‐optimal health condition‐management.

Thus, in addition to addressing the social and political determinants of food insecurity, there is an urgent need to attend to food insecurity as a health issue, and to consider healthcare professionals’ roles in supporting food insecure patients. These issues have been the focus of important work internationally (Berkowitz & Fabreau, 2015; Flores & Amiri, 2019; Kregg‐Byers & Schlenk, 2010; Pooler, Hoffman, & Karva, 2018; Seligman & Berkowitz, 2018), but have received little attention in the UK (Kencana, 2016).

This qualitative study explored health professionals’ views and experiences of supporting people living with both long‐term conditions and food insecurity. The study examined: (a) what healthcare professionals understood about household food insecurity in general terms; and (b) their experiences of and perspectives on support for people who were living with one or more long‐term conditions and who might also be food insecure.

## 2 METHODS

Health professionals with experience in working with people with long‐term conditions were recruited from an NHS Board in north east Scotland on an opt‐in basis via a local NHS interest group and mailed invitations to all GP practices in two local networks. Professionals who expressed interest in taking part in the study were given a detailed information sheet and signed a consent form before taking part. Given time constraints, we did not apply any form of quota sampling or monitoring. Ethics Review Board and NHSR&D management permission were obtained.

A combination of focus groups and semi‐structured interviews was used, in part for pragmatic reasons associated with the challenges of convening focus groups with busy professionals, but also, to harness the different strengths of the two methods. We recognised that focus groups could provide more insight into the norms and variations of clinical practice through peer‐to‐peer discussions, and offer informants an opportunity to learn at the same time (Ritchie, Lewis, Nicholls, & Ormston, 2013). We also appreciated that individual interviews could allow closer investigation of informants’ particular experiences.

Topic guides were developed to ensure coverage of key topics. We opened each interview and group discussion by introducing the definition of food insecurity that underpinned this enquiry. We mentioned four domains of the lived experience of food poverty (Radimer, Olson, & Campbell, 1990) to help prompt reflection and discussion about potentially relevant implications for people with long‐term conditions and for professional work with people affected: involuntary constraints to a sufficient quantity and quality of food; psycho‐social anxiety and uncertainty about food access; and social exclusion.

KM led the focus groups (with support from FD and VE) and conducted the interviews between March and May 2016. Focus group discussions and interviews were audio recorded and transcribed verbatim and transcripts analysed thematically. During the first stage of analysis, all authors read a sample of interview transcripts independently to identify key concepts and potential codes. A draft coding index was drawn up by KM and refined after the authors met again to compare and contrast their readings of further transcripts. All transcripts were coded manually by KM using the refined coding index. The themes reported here were developed in discussion, working by considering data indexed under particular codes and with careful attention to the evident diversity of professional awareness/experience and perspectives.

## 3 FINDINGS

Twenty primary and secondary care health professionals took part in total. Two focus groups were conducted with (a) five community nurses and one hospital‐based nurse, and (b) five consultant physicians. Nine individual interviews were undertaken with general practitioners, public health professionals, a community psychiatric nurse, diabetic nurse specialist, dietitian, health psychologist and nurse manager.

We present our main findings using three main thematic headings. (1) There was *diversity of understanding and experience of food insecurity* among the health professionals (some were much more aware than others). (2) Between them the health professionals identified a range of *negative impacts of food insecurity on condition‐management,* especially for diet dependent conditions or medication regimes, and for mental health. (3) Health professionals can face significant *practical and ethical uncertainty about identifying and responding to food insecure patients* (judgements about whether and how to raise questions about food insecurity with people, tailor dietary advice, and draw boundaries around professional responsibility are not always straightforward).

### 3.1 Diversity of professional understanding and experience with food insecurity

Participants had quite varied levels of awareness and understanding about household food insecurity in general terms, and varied experience of dealing with it in their professional practice. Several acknowledged that our explanatory introduction and in some cases hearing from others in a focus group raised their awareness of the problem, and prompted them to think differently on some patients. For example:

it’s given me food for thought actually in that to think about the fact that people who aren’t nourished, to think is it because they, not that they don’t want, but that they can’t afford. P108

More limited experience of working with food insecure patients could, as some participants reflected, be due to working in geographic areas or professional roles where there were fewer food insecure patients or where food insecurity would have less of an impact on patients’ conditions or condition‐management. But a lack of general awareness and understanding of food insecurity as an issue could also tend to work against recognition that particular patients might be struggling with it.

Those health professionals who reported more experience of working with food insecure patients recognised that while some patients were willing to tell them quite openly about their challenges with money and/or access to food, others might be more sensitive and reluctant to admit financial difficulties and food insecurity:

People are surprisingly honest and will say ‐ y’know sometimes they joke about it ‘cause I think there is a pride about admitting you maybe don’t have enough money at the end of your benefits cheque… They’ll often say to you as well that they’ll go without food and, you know, when you ask “why would that be?” it then tends to come out that… they can’t afford to eat… or they’ll say they’ve used a food co‐op [bank] and you know they can only go to the food bank I think so many times, so you’ll know in desperation they’ve used a food bank, so… that’s kind of how the conversation starts. P111

They would usually say I don’t have enough money, some do, some don’t….keep it secret. But then you can find out in other ways. P113

Although an awareness of the challenges of food insecurity and the possibility that patients might be inclined to cover it up could prompt health professionals to look out for cues that might indicate a problem, the sensitivity and potentially hidden nature of food insecurity is a source of practical and ethical challenge that we return to below.

### 3.2 Negative impacts of food insecurity on health condition‐management

Whether they came with prior understanding or learned about food insecurity via our introductory explanations, all participants recognised (either because they actively held the belief before the study, or because participation in it had prompted them to reflect on their experiences) that it could impact negatively on peoples’ ability to manage health conditions. The problems they had seen or could anticipate in practice related to three main issues. First, food insecurity could be quite directly problematic for conditions influenced by diet. Second, it could impair the efficacy and safety of some medications. Third, the stress associated with food insecurity could impact mental wellbeing and people's capacity to manage other health conditions.

#### 3.2.1 Conditions influenced by diet

Not surprisingly, concerns about the implications of food security for people living with diabetes were mentioned relatively frequently. Some health professionals were acutely aware of patients who were relying on a food bank, and who thus lacked the opportunity to choose and eat the types of foods necessary for good condition‐management. For example:

the other thing about food insecurity is also the types of food that’s on offer through the foodbanks so if somebody is diabetic, what’s on offer? P110

One participant described a person with diabetes whom she had worked with over a long period and knew to be extremely financially vulnerable and chronically food insecure. She had worked with him to help him reduce his blood glucose levels through diet, but found he could not afford to sustain a healthy dietary regime, and (at least in part as a result) needed to start using insulin:

He did end up on insulin and ‐ we did have times when he reduced the carbs and went for healthier options but he said he just couldn’t afford to sustain it and so would go back to the carbs ‐ and so we ended up on insulin to try and combat that P113

Several other informants focused on chronic obstructive pulmonary disorder (COPD) and pointed out how food insecurity could compound other challenges of good nourishment with this condition. People need a relatively high calorie diet to deal with the metabolic demands of COPD (“*they have to aim for 3000 to 4000 calories per day”)*, but can also struggle with condition‐related poor appetite as well as physical weakness and so limited functional mobility and inclination to cook meals. An optimal diet for someone with COPD is relatively expensive, and for some people prohibitively so:

I do think diet and respiratory disease is neglected, and I think people, the most vulnerable people are those who don’t have a good diet. Sometimes I think it is related to income. P108

Some of them are just living on packet crisps. So they don't have any energy. If they eat better, they would have more energy to expand. But it's like a bad circle. Group Discussion

#### 3.2.2 Medication regimes influenced by food

There were also concerns that food insecurity could negatively impact the effectiveness and safety of oral medicines, both by undermining people's scope to take medicines as recommended and because of the role food plays in the pharmacokinetics of oral drug absorption. Participants pointed out that medication timing is often prescribed in relation to meals or snacks, for example before eating, with food or after eating. For food insecure people,

It’s not always possible to take their medication with food, or before or after food sort of given those conditions. I think that has a big impact on how people manage their condition. P111

Several participants also recognised that recommendations to take particular medicines before, with or after food are usually intended to promote effectiveness or avoid unwanted side effects, and that both could be reduced by food insecurity. For example:

from a food point of view it can have an effect. The medication itself can either be more potent or less potent depending on the circumstance….a lot of medications ask to eat with something or before taking the medication because it can irritate stomach linings. Most medications, if not all medications, have side effects and they alone could be amplified. P112

#### 3.2.3 Impacts on mental health and socio‐emotional wellbeing

Informants also expressed concerns about the negative impact that living with food insecurity could have on people's mental health, and that this could have further negative implications for the management of mental health and other health conditions. Some gave specific examples of working with food insecure individuals who had expressed feelings of worthlessness, embarrassment and frustration at their situation,

I suppose it’s gonna have an impact on peoples’ emotional wellbeing because if they’re food insecure, often it’s quite, well I imagine people get quite embarrassed about that and then seeing documentaries about how people get are quite low in mood and all these things have a massive impact on the way people can manage their conditions. P101

A few participants noted that financial worries could negatively impact people's ability to manage their health conditions. This extract, which relates to the financially vulnerable man with diabetes who featured above, illustrates a health professional's observation that a patient's blood glucose levels became substantially raised when he found out he would lose a social security entitlement that was a key element of his household income:

he was also eligible for that bedroom tax that came out and that was a real source of stress for him and his blood sugars went through the roof. P113

### 3.3 Practical and ethical uncertainties about working with food insecure patients

Our interviews revealed significant uncertainty and difference of opinion among health professionals about whether and how they should try to identify and respond to the challenges patients could face with and as a result of food insecurity. In part, of course, this reflected the fact that some health professionals had little awareness or understanding of food insecurity in general (Theme 1 above). However, there was also a distinct lack of consistency and clarity of views about whether, why and how health professionals should take responsibility and engage with the problem.

At one end of the spectrum, a few informants appeared unconcerned about food insecurity, either regarding it as ‘normal’ for the people affected or, at least sometimes irrelevant for their own work with them.

We're not looking at it, like food insecurity, because people don't necessarily seem anxious about it because that's how they've always lived. Group Discussion

It didn't really impact on how I worked with her. P101

And we heard little or no evidence that professional teams had focused on food insecurity in discussions oriented to professional development or practice improvement.

I think we discuss it vaguely in that you know someone will maybe say “I felt really sorry for that patient” or “how could you live in those circumstances?” and XYZ but I don’t know that we set out to have discussions about food poverty like we would have a discussion about diabetes or heart disease or irritable bowel syndrome. P111

Overall, however, health professionals acknowledged at least some need to understand and respond to food insecurity among the people they served. They mentioned professional responsibilities, for example, to be aware of their patients’ circumstances, to tailor advice appropriately, to provide emotional support, and to work effectively with other agencies and services with relevant financial, food and broader social remits. Still, there were significant questions and differences of opinion about what exactly was appropriate to discuss, and why greater familiarity with the complex causes and implications of food insecurity, might add to as well as help resolve the uncertainty health professionals experience when dealing with particular situations.

#### 3.3.1 Identifying patients for whom food insecurity is an issue

When patients do not overtly mention or provide clear clues that they have difficulties relating to food insecurity, health professionals can be disinclined and/or unsure how to ask to find out. Our informants were aware that potential problems with money and access to food were *“a very sensitive topic”* (P110) and some thought some patients would assume health professionals would judge them negatively or otherwise inappropriately if they recognised they were food insecure.

One informant suggested in a focus group that some sort of a protocol or screening tool could help raise the issue of food insecurity, and we asked a few others about the proposal in subsequent interviews. Perceived advantages included that it could be a “*softer way to broach that subject”* and that if used routinely it could serve to establish a profile of food insecurity in the practice (or service), which could raise awareness among professional staff and inform the clinical management of affected patients. The only note of caution raised in these conversations was that it would need to be *“quick and easy to use”* (but see Discussion below).

#### 3.3.2 Working appropriately with patients for whom food insecurity is an issue

Some informants highlighted the importance of signalling respectfully to their patients, that they were aware they were dealing with food insecurity:

you’ve got to show understanding of where they are in terms of their resource as to what they have. P113

However, some also recognised the difficulty of communicating sensitively from their more economically comfortable professional positions, noting potential for erroneous assumptions about what food insecurity was like:

We’re in paid income and we also have different expectations and some of our patients, that’s what they’ve always known. Group Discussion

People will think ‘you don’t know anything about me’. P110

Most of the discussion about how to work well with people who were food insecure focused on practical responses to challenges relating to food and condition management.

A few informants indicated that they or colleagues were giving people dietary and other lifestyle advice which was unaffordable or otherwise unrealistic in their circumstances. One in particular lamented that patients’ financial capability to do what was generally recommended on the basis of evidence was rarely considered in routine clinical practice. These informants noted a need to be aware of both the costs of food and the means people had to prepare them.

Sometimes people came from a really deprived area of town and were on really limited income. Trying to give them practical advice on how to load up in a calorie perspective in a way that was inexpensive was problematic. P108

You’ve got to look at what can you eat it on. Have you got a microwave and if you haven’t, have you got a kettle? Group Discussion

However, there could be a tension between tailoring advice to make it more realistically achievable and wanting to ensure that food insecure patients were not deprived of the opportunity to benefit from best evidence‐based recommendations.

#### 3.3.3 Working with other services and agencies

Several informants emphasised that it was not up to health professionals to ‘solve’ food insecurity for patients. Some did however, accept a responsibility to refer people in need on to other organisations that were better placed to help address underlying issues.

if I think that food poverty is part of the problem that your diet isn’t great; I need to signpost you to someone who can help fix that. … As opposed to me fix it. P108

For help with financial matters and income maximisation, a so‐called Cash in Your Pocket scheme, and Citizens Advice Bureaux were mostly commonly cited, along with referrals to social workers. However, some informants noted that shame and embarrassment could deter people from acting on their referrals.

Some informants also mentioned the possibility of referring people to food banks, but most were unsure how to do this, and only one informant reported actually making such a referral. When informants thought that poor nutritional knowledge and cooking skills were exacerbating food insecurity, they considered it useful to be able to signpost people to cooking classes, but sometimes had questions about the appropriateness of available classes.

## 4 DISCUSSION

This exploratory, qualitative study with health professionals identified a wide range of levels of awareness of food insecurity and experience of engaging with food insecure patients. At least after being introduced to the concept of food insecurity, the health professionals could think of several ways in which it could impact adversely on people's ability to manage their long‐term health conditions. However, there was significant difference of opinion and uncertainty about what they should do to identify and address food insecurity in the context of their clinical practice.

We focus this discussion primarily on the practical and ethical concerns raised by participants, considering how food insecurity is perceived in terms of its relevance to health professionals’ business; the social sensitivities surrounding recognising food insecurity in practice; how health professionals are responding to food insecurity to improve condition‐management and well‐being; and the educational and research implications of this work.

### 4.1 Food insecurity as health professionals’ business

We were concerned that some health professionals appeared to accept that food insecurity was ‘normal’ for some patients and not something they needed to change their clinical practice to address. Their comments may reflect a sense that little can be done currently to reduce food insecurity and/or an interpretation that food insecurity is just a new label for some of the longstanding problems of poverty that their clinical practice is already sensitive to. If, however, their comments reflect a lack of concern for social justice and/or a lack of awareness of how patients’ social circumstances can affect their scope to contribute to their healthcare, they are out of kilter with research‐informed policy commitments in Scotland and elsewhere to address problems of poverty within healthcare to improve people's ability to manage and live well with long‐term health conditions (Berkowitz & Fabreau, 2015; Entwistle, Cribb, & Owens, 2018; Kregg‐Byers & Schlenk, 2010; Lindberg, Lawrence, Gold, Friel, & Pegram, 2015; Moscrop & MacPherson, 2014; NHS Health Scotland, 2015, 2016; Patil et al., 2017).

### 4.2 Social sensitivities and the recognition of food insecurity

Some health professionals were notably uncertain and anxious about raising the issue of food insecurity with patients who did not voluntarily disclose problems. They feared their inquiries causing offence or their responses otherwise getting it wrong. Similar professional discomfort has been highlighted in other high‐income country contexts (Barnidge, LaBarge, Krupsky, & Arthur, 2017; McGee, 2018; Moscrop & MacPherson, 2014). It reflects a well‐founded appreciation that many people are reluctant to disclose their financial situation (especially difficulties), including with healthcare professionals (Barnidge et al., 2017; Cullen, Woodford, & Fein, 2019). Poverty is socially stigmatising and food insecurity, especially when it leads to food bank use, is often accompanied by shame and embarrassment (Douglas, Sapko, Kiezebrink, & Kyle, 2015; Purdam, Garratt, & Esmail, 2016; van der Horst, Pascucci, & Bol, 2014).

The positive interest that health professionals expressed in a screening tool to help them identify cases of food insecurity is perhaps not surprising given their ‘how to’ uncertainty. But such a tool may not be such a good solution as it first seems. Depending on its sensitivity and the discreteness of its administration and use, a screening tool would not necessarily deal well with patients’ concerns and could indeed generate harms in the form of patient anxiety, shame and alienation from health service providers (Easton, Entwistle, & Williams, 2013). Also, the justification for screening rests on the improvements to clinical management that would follow (Berkowitz & Fabreau, 2015; Elwell‐Sutton, Marshall, Bobby, & Volmert, 2019; Silverman et al., 2015), and these are currently unclear, especially when the salient comparator is clinical management by health professionals who get to know about their patients’ food insecurity via supportive and non‐judgemental conversations. Practical experiences with existing food security screening tools (Gundersen, Engelhard, Crumbaugh, & Seligman, 2017; Pooler et al., 2018; Thomas, Fitzpatrick, Sidani, & Gucciardi, 2018) need careful investigation before any widespread use.

### 4.3 Responding to food insecurity to improve condition‐management and well‐being

The problems that the health professionals in this study suggested that food insecurity could cause for patients’ management of their long‐term conditions were broadly consistent with much research evidence about the implications of diet for health, and with the growing literature about the coping strategies and experiences of people who are food insecure (Aibibula et al., 2017; Bomberg, Neuhaus, Hake, Engelhard, & Seligman, 2018; Kalichman et al., 2015). These problems seem set to increase as more people in the UK are developing and living with often multiple health conditions, food costs are predicted to continue to rise and key groups become yet more financially vulnerable (Jones, Conklin, Suhrcke, & Monsivais, 2014; Josepeh Rowntree Foundation, 2018). The need for awareness and vigilance among those working in the healthcare system to support people with their condition self‐management has never been more acute (Loopstra, 2018; Loopstra et al., 2019).

Yet beyond a general awareness that good health condition‐management and support requires attention to the complex social and economic determinants underpinning people's lives and health conditions (Elwell‐Sutton et al., 2019), the questions of what should be done, how and by whom within clinical practice are far from well answered. Some clinical interventions will at best be partial and temporary ameliorations ‐ the proverbial sticking plasters on gaping wounds ‐ and most raise some cause for concern as well as some hope for benefit.

For example, referrals from health professionals to food banks might secure a little more food than some patients would get otherwise. But food supplied through food banking systems is often insufficient in to meet clients’ nutritional needs, either quantitatively or qualitatively (Bazerghi, McKay, & Dunn, 2016; Cook, 2017; Galesloot, McIntyre, Fenton, & Tyminski, 2012; Gany et al., 2013; Irwin, Ng, Rush, Nguyen, & He, 2007; Ross, Campbell, & Webb, 2013), and even when food bank operators are aware of and attempt to meet clients’ particular nutritional requirements, they can rarely do so with their available resources (Douglas, Ejebu, et al., 2015). The stigma and challenge to self‐respect of food bank use remains for patients, and health professionals might reasonably worry about implicitly supporting the long‐term use of a short‐term measure (Beck, 2019; McIntyre, Tougas, Rondeau, & Mah, 2016).

Our analysis also surfaced a tension health professionals can experience when they realise that clinical recommendations based on what is generally effective advice will be impossible for some people to follow due to cost, but adjusting recommendations to reflect something more manageable seems to make for second best care for poorer patients. Either way, there are concerns about increasing health inequities for already vulnerable people (Lorenc, Petticrew, Welch, & Tugwell, 2013; White, Adams, & Heywood, 2009) and significant scope for contestation about how balances should be struck when trying to respectfully enable people to live well with long‐term conditions (Entwistle, Cribb, Watt, et al., 2018).

### 4.4 Study limitations and strengths

The obvious limitations of the study are the relatively small number of healthcare professionals who could be recruited and interviewed in our time frame, and the rather pragmatic combination of focus groups and interviews. It is possible that health professionals with more experience of working with very poor patients were more likely to take part, but we were particularly keen to hear about experiences with food insecure patients. Therefore, we can make no claims about the *distribution* of professional understandings and experiences reported here. In addition, although some points seemed to be recurring, we do not claim full data saturation. Qualitatively, however, we have been able to highlight several practical and ethical concerns that at least some health professionals need support with, and that warrant further attention.

### 4.5 Guidance and support for health professionals

Our study suggests a need for support for health professionals to better understand the challenges of food insecurity as experienced by people with long‐term health conditions, and to develop respectful and effective approaches to working to enable them to manage and live well with their conditions. The perspectives of people who live with(or have lived with) food insecurity will be crucial here.

## 5 CONCLUSION

Food insecurity impacts significantly on people's ability to manage and live well with long‐term health conditions. Health professionals need to be able to support people who are food insecure appropriately, but there are currently practical and ethical uncertainties about the appropriateness of some proposed strategies for identifying and responding to cases involving food insecurity in practice. Without denying the need to address the root causes of food insecurity, we suggest that a programme of research and development is needed to ensure that health systems foster good professional practice in this area and contribute effectively with social services and other agencies to support the current casualties. Such a programme needs to be conducted in partnership with people directly affected by food insecurity.
